# Laparoscopic Distal Pancreatectomy for Lymph Node Metastasis around Splenic Artery from Hepatocellular Carcinoma in a Patient with Portal Annular Pancreas

**DOI:** 10.70352/scrj.cr.24-0130

**Published:** 2025-04-11

**Authors:** Kyosuke Habu, Shintaro Akamoto, Shin Imura, Yuta Fujiwara, Yusuke Konishi, Tetsuji Fukuhara, Kazuhiko Nakagawa, Keiichi Okano

**Affiliations:** 1 Department of Surgery, Sumitomo Besshi Hospital, Niihama, Ehime, Japan; 2 Department of Gastroenterological Surgery, Faculty of Medicine, Kagawa University, Miki, Kagawa, Japan

**Keywords:** lymph node metastasis from HCC, portal annular pancreas, laparoscopic distal pancreatectomy

## Abstract

**INTRODUCTION:**

Lymph node metastases after hepatocellular carcinoma (HCC) resection exist, although they are not common. However, solitary metastasis to the splenic artery lymph node with suspected pancreatic invasion after HCC resection is rare. In certain cases, surgical resection is performed to improve patient outcomes. We report a case of lymph node metastasis resected by laparoscopic distal pancreatectomy (LDP) in a patient with a unique anatomical anomaly known as portal annular pancreas (PAP).

**CASE PRESENTATION:**

A 79-year-old Japanese man underwent laparoscopic left lateral segmentectomy for HCC. Two months after the surgery, alpha-fetoprotein levels remained elevated. Plain computed tomography revealed a swollen lymph node along the splenic artery involving the pancreas and the PAP. We suspected a solitary metastasis to the lymph node around splenic artery with pancreatic invasion. LDP was performed for complete resection of lymph node metastasis. Although the patient developed a grade B postoperative pancreatic fistula, he was discharged on postoperative day 33 under conservative treatment of antibiotics. He has remained recurrence-free for 4 years and 3 months after surgery.

**CONCLUSIONS:**

LDP was successfully performed for lymph node metastasis around the splenic artery in an HCC patient and resulted in long-term survival. Surgeons should be aware of the unique anatomical characteristics of PAP during LDP.

## Abbreviations

CT
: computed tomography
HCC
: hepatocellular carcinoma
LDP
: laparoscopic distal pancreatectomy
LN
: lymph nodes
LNM
: Lymph node metastasis
PAP
: portal annular pancreas
POPF
: postoperative pancreatic fistula
SA
: splenic artery
SV
: splenic vein

## INTRODUCTION

Lymph node metastases (LNMs) after hepatocellular carcinoma (HCC) resection are not frequent, but are not uncommon. However, solitary metastasis to the splenic artery (SA) lymph node (LN) with suspected pancreatic invasion after HCC resection is rare. In certain cases, surgical resection is performed to improve patient outcomes.

Of note, the patient had a unique anatomical anomaly known as the portal annular pancreas (PAP). PAP is an asymptomatic congenital pancreatic anomaly in which the portal and/or mesenteric veins are encased within pancreatic tissue.^1)^ Understanding the anatomical characteristics of PAP and its associated risk of postoperative pancreatic fistula (POPF) is crucial for surgeons.

## CASE PRESENTATION

**Fig. 1:**
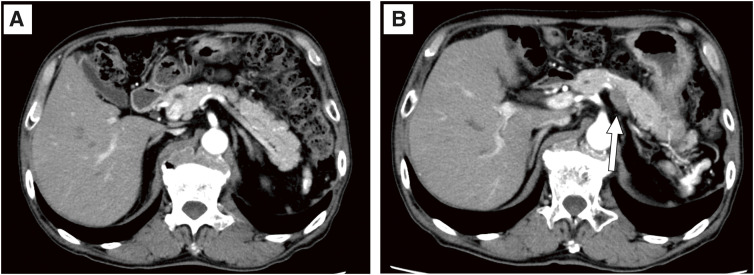
(**A**) No lymph node metastasis near the splenic artery or pancreas was detected prior to the initial surgery. (**B**) Two months after the initial surgery, CT revealed an enlarged lymph node measuring 30 × 20 mm adjacent to the splenic artery and pancreas (white arrow). CT, computed tomography

**Fig. 2:**
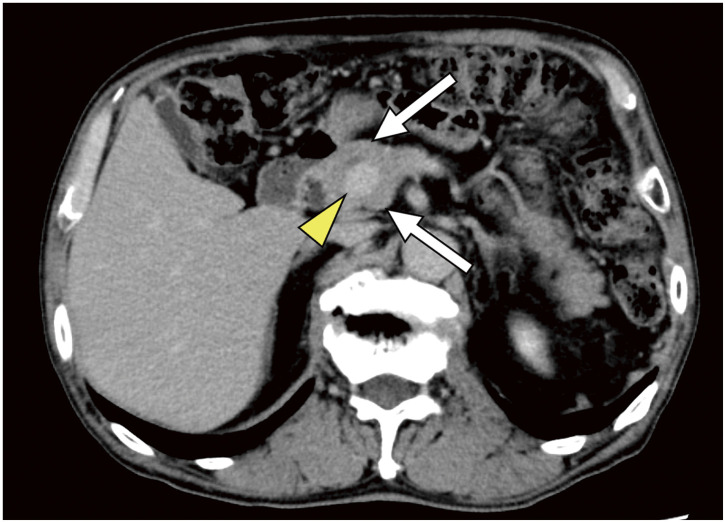
The yellow arrowhead shows the portal vein and the white arrow shows the pancreatic parenchyma. The portal vein is surrounded by the pancreas, a condition known as portal annular pancreas.

A 79-year-old Japanese man presented with hepatitis B virus-related cirrhosis, diabetes, hypertension, and rheumatoid arthritis. Plain computed tomography (CT) revealed two multinodular confluent-type HCCs in segments S2 and S3 of the liver, measuring 15 × 15 mm and 19 × 20 mm, respectively, with a total transverse diameter of 35 mm. The patient underwent laparoscopic left lateral segmentectomy. Pathological examination revealed moderately differentiated HCC, with tumors measuring 28 and 20 mm in diameter. According to the 8th edition of the Union for International Cancer Control Tumor–Node–Metastasis staging, the pathological diagnosis was T2N0M0 stage II. No vascular invasion was found. After hepatectomy was performed, the patient was followed up as an outpatient. The preoperative AFP level was 1,328 ng/mL; however, 2 months postoperatively, the AFP level did not normalize and remained elevated at 756 ng/mL. Laboratory data are shown in **Table 1**. Enhanced CT revealed a 30 × 20 mm swollen LN abutting the SA and pancreas, which had not been detected preoperatively (**Figs. 1A** and **1B**). No LNM near the SA or pancreas was detected prior to the initial surgery. CT also revealed PAP and demonstrated that the tumor was in contact with approximately half of the circumference of the SA and the pancreas (**Fig. 2**).

**Table 1:** The patient’s laboratory results 2 months after lateral hepatic segmentectomy

Parameter	Value	SI Unit	Parameter	Value	SI Unit
WBC	3750	10^3/µL	BUN	23.2	mg/dL
HGB	13.0	g/dL	Cre	0.79	mg/dL
PLT	11.1	10^4/µL	PT	11.7	s
			PT (INR)	0.94	
AST	14	IU/L	APTT	30.7	s
ALT	12	IU/L			
γ-GTP	12	IU/L	AFP	756	ng/mL
T-Bil	0.67	mg/dL	PIVKA-2	124	mAU/mL
D-Bil	0.18	mg/dL	CEA	1.5	ng/mL
AMY	109	IU/L	CA19-9	<2.0	IU/mL
Lipase		IU/L			
CRP	0.01	mg/dL	HbA1c	5.5	%

WBC, white blood cell; HGB, hemoglobin; PLT, platelet; AST, aspartate transaminase; ALT, alanine transaminase; γ-GTP, gamma-glutamyl transpeptidase; T-Bil, total bilirubin; D-Bil, direct bilirubin; AMY, amylase; CRP, C-reactive protein; BUN, blood urea nitrogen; Cre; creatinine; PT, prothrombin time; PT (INR), prothrombin time (international normalized ratio); APTT, activated partial thromboplastin time; AFP, alpha fetoprotein; PIVKA-2, protein induced vitamin k absence or antagonist-II; CEA, carcinoembryonic antigen; CA19-9, cancer antigen 19-9; HbA1c, glycated hemoglobin

The portal vein was found within the uncinate process with a normal anteportal main pancreatic duct. Fusion of the pancreatic body and uncinate process occurred at the splenoportal confluence.

**Fig. 3:**
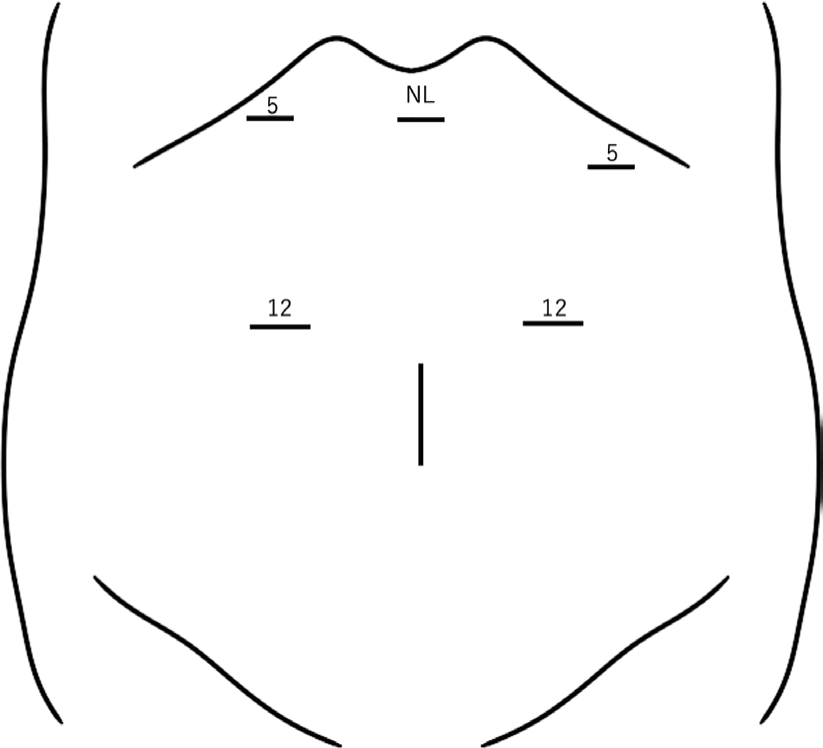
The ports are arranged as shown. NL, Nathanson liver retractor

**Fig. 4:**
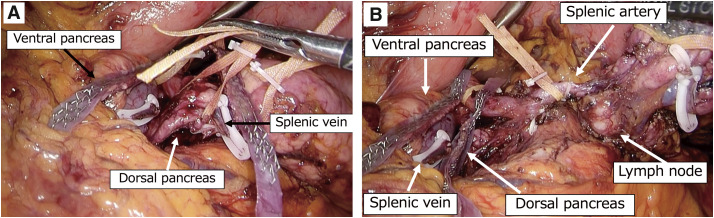
(**A**) After dividing the ventral pancreas. (**B**) After dividing the pancreas twice, the splenic artery is taped.

**Fig. 5:**
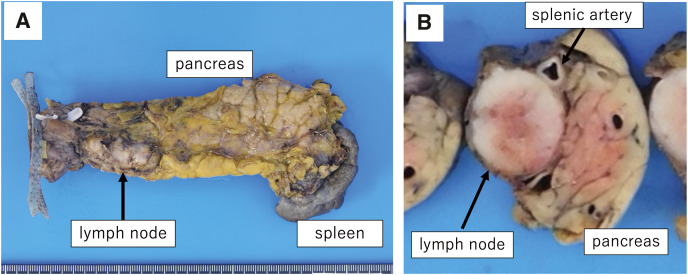
(**A**) Extracted specimens. (**B**) After sectioning. The enlarged lymph nodes are located along the splenic artery and abutment of the pancreas. Lymph nodes are removed without damaging the capsule.

**Fig. 6:**
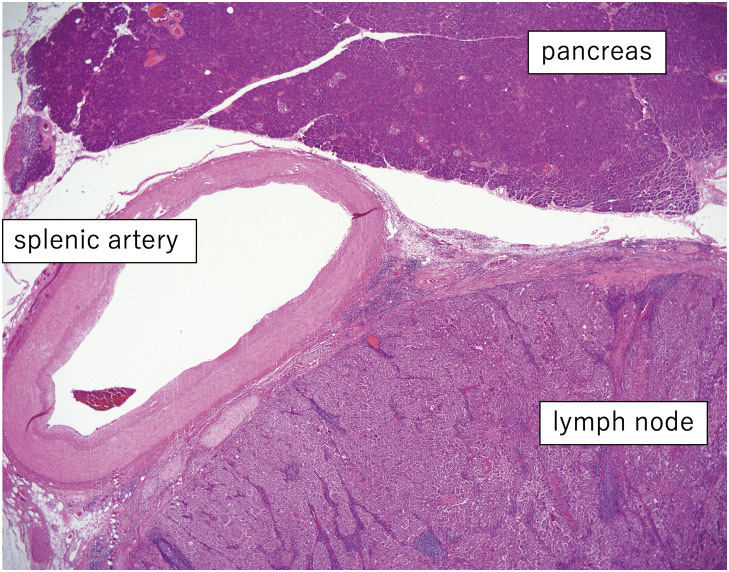
The pathological findings are similar to those of previous hepatocellular carcinoma operations. Although the lymph nodes are in contact with the splenic artery and the pancreatic parenchyma extensively, no histological evidence of invasion is observed.

We diagnosed a solitary metastasis to the LN around SA with pancreatic invasion and decided to perform an LDP. Under general anesthesia, the patient was placed in the lithotomy position. The port placement is shown in **Fig. 3**. After the greater omentum was widely opened toward the spleen, the left gastroepiploic vessels were clipped and divided. The stomach was suspended using 2-0 nylon sutures and retracted using a Nathanson liver retractor. The common hepatic, gastroduodenal, and splenic arteries were exposed and taped. During surgery, LNM was found in close proximity to the SA and pancreas, raising concerns about potential invasion. In addition, the splenic vein (SV), superior mesenteric, and inferior mesenteric veins were identified. The ventral pancreatic parenchyma was taped and elevated from the anterior aspect of the portal vein. Next, the ventral pancreas was divided using the Endo GIA reinforced black reload with Tri-Staple 60 mm (COVIDIEN, North Haven, CT, USA). We adopted the slow parenchymal flattening technique^2)^ to prevent POPF. The PAP surrounding the portal vein, which had been suspected preoperatively, was identified (**Fig. 3**). The SV and pancreatic dorsal artery were clipped and later divided. Dorsal pancreatic dissection was performed to insert the stapler, which was used to divide the dorsal side of the PAP (**Fig. 4**). Finally, the SA was divided after clipping, and additional mobilization of the pancreas and retroperitoneum was performed to retrieve the specimen. Surgery time was 246 minutes and blood loss was up to 100 mL. The resected specimens are shown in **Fig. 5**. The pathology was consistent with that of HCC metastasis (**Fig. 6**). Although intraoperative findings showed that the LNs were severely adherent to the SA and pancreatic parenchyma, no histological evidence of invasion was observed. Postoperatively, a grade B pancreatic fistula was observed, which resolved mildly with conservative treatment. The patient was discharged on postoperative day 33. No exacerbation of postoperative glucose intolerance was observed. Four years and 3 months after the surgery, the patient is still alive and there has been no recurrence or increase in the tumor marker levels.

## DISCUSSION

In 2020, primary liver cancer was the sixth most diagnosed cancer and the third leading cause of cancer-related deaths globally.^3)^ HCC is a significant global health challenge and accounts for approximately 90% of primary liver cancers.^4)^ Surgical resection offers the best potential for cure in HCC among HCC treatment strategies; however, HCC is often diagnosed at an advanced stage, making surgery infeasible. Moreover, even after curative resection, HCC has a high recurrence rate, ranging from 54% to 82.6%.^5–8)^ The prognosis worsens significantly when extrahepatic metastases develop after resection, with the incidence of such metastases ranging from 9.8% to 19.3%.^7–9)^ The lungs are the commonest site for extrahepatic metastases of HCC, followed by the bones, LNs, peritoneum, adrenal glands, and the brain.^9,10)^ The prevalence of LNMs in patients with HCC ranges from 0.3% to 5.9%,^11–15)^ with higher rates observed in autopsies (25.5%–32%).^10,16,17)^

The most frequent sites of LNM are the regional LNs, followed by the intra- and extra-abdominal LNs.^15)^ Among these, the retroperitoneal^18)^ and hilar.^19,20)^ LNs are most commonly involved, while para-aortic.^21)^ LNM is less common. Notably, there are no previous reports of SA LNM as observed in this case. Typically, LNM of HCC follows the natural lymphatic drainage from the hepatic portal vein to the peripancreatic and periaortic LNs (96%). However, in some cases, LNM occurs in the peripancreatic and periaortic nodes without involvement of the hepatic portal vein nodes, a phenomenon known as skip metastasis (4%).^22)^

Cirrhosis can lead to obstruction of capillary lymphatics and the formation of collateral lymphatic pathways owing to connective tissue accumulation. It also results in increased portal lymphatic flow^23)^ and heightened lymphangiogenesis.^24,25)^ Altered lymphatic flow due to cirrhosis has been linked to nonsystematic LNM.^26,27)^

In this case, the presence of cirrhosis and prior left lateral segmentectomy for HCC likely altered the lymphatic flow, leading to distal SA LNM rather than proximal LN involvement.

LNMs in HCCs are rare and are generally associated with poor prognosis.^28)^ Although distant LNM is also rare, it should be considered in patients with elevated levels of tumor markers. It can be diagnosed with CT, biopsy, or positron emission tomography-CT.^29)^ Most extrahepatic metastases of HCC occur at multiple sites, precluding surgical resection and limiting treatment options. Thus, early detection of recurrence and the establishment of a rigorous follow-up system are crucial.

Although there is no consensus on the optimal approach for extrahepatic metastases of HCC, treatment options include surgery, radiotherapy, and chemotherapy.^13,18,19,28,30–34)^ Resection of metastatic LNs offers a promising prognosis and may benefit selected patients.^12,33)^ The indication for surgery for LNM of HCC is a localized disease that can be safely resected without any residual tumor.

In this case, resection was applied because of the solitary nature of LNM and the absence of other intrahepatic metastases. Preoperative CT imaging revealed enlarged LNs along the SA, close to the pancreas. Endoscopic ultrasound or magnetic resonance imaging might have been useful for accurately assessing preoperative vascular invasion by LNMs. However, the definitive determination of invasion was made intraoperatively. Due to concerns about potential invasion, simple enucleation of the LN alone was deemed challenging. To ensure an oncologically safe resection while preserving the LN capsule, a combined pancreatic resection was considered to be the most appropriate approach. We believe that LDP is an effective treatment option for lymph node metastases along the splenic artery with suspected pancreatic invasion after laparoscopic HCC surgery. While there has been a report of LDP for splenic hilum LNM from lung cancer,^35)^ to our knowledge, there are no reports of laparoscopic resection for recurrent LNM after HCC surgery. This case highlights the feasibility and potential benefits of laparoscopic resection for LNM in selected patients, particularly in the context of HCC recurrence. As minimally invasive liver resection is rapidly increasing, there will be an increasing number of opportunities to consider minimally invasive surgery for recurrent lesions of HCC.

In addition, the present case involved a PAP, necessitating careful attention to anatomical variations. PAP occurs in 0.8%–2.5% of cases,^36–38)^ but the true frequency is likely to be underreported owing to its subtlety in diagnosis. PAPs are classified in two ways. Karasaki et al.^37)^ classify the SV into three types based on its position: suprasplenic, infrasplenic, and mixed. Meanwhile, Joseph et al.^39)^ classify PAP into three types based on the relationship between the pancreatic duct and the portal vein: type 1, ventral bud of the pancreas fuses with the body and ductal system of the pancreas posterior to the portal vein; type II, association with the pancreas divisum; and type III, only the uncinate process is involved in vessel encasement and fusion. Using Kawasaki and Joseph’s classification, this case was a mixed and ante-portal duct type (type III), respectively. The frequency of the mixed anteportal type is 2%,^36)^ making it very rare. Reports of pancreatic resection for PAP are relatively rare but have been sporadically documented. The reported cases of distal pancreatectomy in patients with PAP are listed in **Table 2**.^1,40–47)^ Laparoscopic surgery was performed in only four cases.

**Table 2:** Summary of cases of distal pancreatectomy in patients with portal annular pancreas (PAP)

Author	Year	Age	Sex	Pancreatic disease	Karasaki’s classification	Joseph’s classification	Laparoscopic surgery	No. of cut margins	POPF
Hashimoto^40)^	2009	39	F	MCN	Suprasplenic	I	No	2	Yes (grade A)
Jang^41)^	2012	74	F	IPMN	Mixed	–	Yes	2	Yes (grade A)
Yamaguchi^42)^	2013	80	F	Pancreas sarcoidosis	Suprasplenic	I	No	2	No
Harnoss^1)^	2014	48	F	Suprarenal cancer	Suprasplenic	III	No	2	Yes (grade B)
Ohtsuka^43)^	2017	63	M	PNET	Suprasplenic	III	No	2	No
Ohtsuka^43)^	2017	61	F	PDAC	Suprasplenic	III	No	2	Yes (grade B)
Yuan^44)^	2017	74	M	PDAC	Infrasplenic	III	No	2	No
Kuriyama^45)^	2020	47	F	SCN	Suprasplenic	III	Yes	2	Yes (grade B)
Abe^46)^	2021	70	M	Pancreatic cancer	–	II	No	2	No
Polyakov^47)^	2023	33	F	Solid pseudopapillary tumor	Suprasplenic	III	Yes	2	Yes (grade B)
Our case	2024	79	M	HCC LNM	Mixed	III	Yes	2	Yes (grade B)

PAP, portal annular pancreas; POPF, postoperative pancreatic fistula; MCN, mucinous cystic neoplasm; IPMN, intraductal papillary mucinous neoplasm; F, female; M, male; PNET, pancreatic neuroendocrine tumor; PDAC, pancreatic ductal adenocarcinoma; SCN, serous cystic. neoplasm; LNM, lymph node metastasis; HCC, hepatocellular carcinoma

The pancreatic division line should be considered when performing distal pancreatectomy in patients with PAP. In PAP, whether the pancreas should be divided at the boundary between the portal vein and superior mesenteric vein (pancreatic isthmus) resulting in two cut margins or on the distal side of the PAP resulting in a single cut margin, remains controversial.

Mendoza et al.^48)^ concluded that a pancreatic tumor with a diameter ≥12 mm is associated with a higher risk of POPF than one measuring <12 mm. Pancreatic thickness in pancreatectomy was also discussed in a review by Pandrowala et al.,^49)^ who stated that it is better to separate the ventral and dorsal pancreas individually than to separate them using a single stapler because of the increased thickness of the distal pancreatic parenchyma.

In the present case, the thickness of the distal side of the PAP was 20 mm, exceeding 12 mm, and it was preferable to divide the pancreas at the isthmus to avoid the risk of a pancreatic fistula. In patients with PAP, increased pancreatic transection may increase the risk of POPF. Unfortunately, in this case, a POPF occurred, but the patient was successfully treated by leaving the drain in place slightly longer than usual and administering antibiotics.

## CONCLUSION

Herein, we reported a case of laparoscopic distal pancreatectomy with portal annular pancreas for splenic artery lymph node metastasis of hepatocellular carcinoma after hepatic resection. Lymph node metastasis is relatively rare in hepatocellular carcinoma but can occur, and resection is effective for isolated metastases. When performing a pancreatic resection, it is essential to consider the presence of PAP on preoperative imaging. In PAP cases, the anatomical relationship between the splenic vein and the main pancreatic duct may vary, and there is an increased risk of postoperative pancreatic fistula.

## ACKNOWLEDGMENTS

The authors thank all parties who assisted in the preparation of this paper. They also thank Editage (www.editage.jp) for the English language editing.

## DECLARATIONS

### Funding

No specific funding was received for this study.

### Authors’ contributions

KH conceived the study, performed the surgical procedure, and drafted the initial manuscript.

SA and KO contributed to the study design, performed the surgical procedure, provided critical revisions to the manuscript, and contributed to the interpretation of the findings.

SI, YF, TF, and KN were involved in performing the surgical procedure and critically reviewed the manuscript.

All authors have read and approved the final manuscript.

Furthermore, all authors agree to be accountable for all aspects of the work, ensuring that any questions related to the accuracy or integrity of the work are appropriately addressed and resolved.

### Availability of data and materials

The datasets and materials used in this study are available upon reasonable request.

### Ethics approval and consent to participate

The study was approved by the Institutional Review Board (Approval No.: S-0001). Informed consent was obtained from the participant prior to their inclusion in the study.

Informed consent was obtained from the participant prior to their involvement in the study.

### Consent for publication

Informed consent was obtained from the patient for the publication of this case report and accompanying images.

### Competing interests

The authors declare that there are no competing interests to report.

## References

[1] Harnoss JM Harnoss JC Diener MK Portal annular pancreas: a systematic review of a clinical challenge. Pancreas 2014; 43: 981–6. 25207658 10.1097/MPA.0000000000000186 PMC4175015

[2] Okano K Kakinoki K Suto H Slow parenchymal flattening technique for distal pancreatectomy using an endopath stapler: simple and safe technical management. Hepatogastroenterology 2010; 57: 1309–13. 21410078

[3] Sung H Ferlay J Siegel RL Global cancer statistics 2020: GLOBOCAN estimates of incidence and mortality worldwide for 36 cancers in 185 countries. CA Cancer J Clin 2021; 71: 209–49. 33538338 10.3322/caac.21660

[4] Galle PR Forner A Llovet JM EASL clinical practice guidelines: management of hepatocellular carcinoma. J Hepatol 2018; 69: 182–236. 29628281 10.1016/j.jhep.2018.03.019

[5] Tabrizian P Jibara G Shrager B Recurrence of hepatocellular cancer after resection: patterns, treatments, and prognosis. Ann Surg 2015; 261: 947–55. 25010665 10.1097/SLA.0000000000000710

[6] Nagao T Inoue S Yoshimi F Postoperative recurrence of hepatocellular carcinoma. Ann Surg 1990; 211: 28–33. 1688488 10.1097/00000658-199001000-00005 PMC1357889

[7] Ochiai T Ikoma H Okamoto K Clinicopathologic features and risk factors for extrahepatic recurrences of hepatocellular carcinoma after curative resection. World J Surg 2012; 36: 136–43. 22051887 10.1007/s00268-011-1317-y

[8] Tanaka K Shimada H Matsuo K Clinical features of hepatocellular carcinoma developing extrahepatic recurrences after curative resection. World J Surg 2008; 32: 1738–47. 18463920 10.1007/s00268-008-9613-x

[9] Kanda M Tateishi R Yoshida H Extrahepatic metastasis of hepatocellular carcinoma: incidence and risk factors. Liver Int 2008; 28: 1256–63. 18710423 10.1111/j.1478-3231.2008.01864.x

[10] Tobe T Endo Y Hattori N Primary liver cancer in Japan. Clinicopathologic features and results of surgical treatment. Ann Surg 1990; 211: 277–87. 2155591 PMC1358432

[11] Yang Y Nagano H Ota H Patterns and clinicopathologic features of extrahepatic recurrence of hepatocellular carcinoma after curative resection. Surgery 2007; 141: 196–202. 17263976 10.1016/j.surg.2006.06.033

[12] Tomimaru Y Wada H Eguchi H Clinical significance of surgical resection of metastatic lymph nodes from hepatocellular carcinoma. Surg Today 2015; 45: 1112–20. 25205550 10.1007/s00595-014-1028-8

[13] Kobayashi S Takahashi S Kato Y Surgical treatment of lymph node metastases from hepatocellular carcinoma. J Hepatobiliary Pancreat Sci 2011; 18: 559–66. 21331804 10.1007/s00534-011-0372-y

[14] Sun HC Zhuang PY Qin LX Incidence and prognostic values of lymph node metastasis in operable hepatocellular carcinoma and evaluation of routine complete lymphadenectomy. J Surg Oncol 2007; 96: 37–45. 17345597 10.1002/jso.20772

[15] Lee BM Choi JY Seong J. Efficacy of local treatment in lymph node metastasis from hepatocellular carcinoma. Liver Cancer 2023; 12: 218–28. 37767066 10.1159/000529201 PMC10521325

[16] Yuki K Hirohashi S Sakamoto M Growth and spread of hepatocellular carcinoma. A review of 240 consecutive autopsy cases. Cancer 1990; 66: 2174–9. 2171748 10.1002/1097-0142(19901115)66:10<2174::aid-cncr2820661022>3.0.co;2-a

[17] Watanabe J Nakashima O Kojiro M. Clinicopathologic study on lymph node metastasis of hepatocellular carcinoma: a retrospective study of 660 consecutive autopsy cases. Jpn J Clin Oncol 1994; 24: 37–41. 8126919

[18] Une Y Misawa K Shimamura T Treatment of lymph node recurrence in patients with hepatocellular carcinoma. Surg Today 1994; 24: 606–9. 7949768 10.1007/BF01833724

[19] Furukawa S Ide T Ito K Treatment strategy for lymph node metastasis of hepatocellular carcinoma using an ICG navigation system: a case report. Surg Case Rep 2023; 9: 211. 38047972 10.1186/s40792-023-01790-w PMC10695890

[20] Uehara K Hasegawa H Ogiso S Skip lymph node metastases from a small hepatocellular carcinoma with difficulty in preoperative diagnosis. J Gastroenterol Hepatol 2003; 18: 345–9. 12603540 10.1046/j.1440-1746.2003.02795.x

[21] Ueda J Yoshida H Mamada Y Surgical resection of a solitary para-aortic lymph node metastasis from hepatocellular carcinoma. World J Gastroenterol 2012; 18: 3027–31. 22736929 10.3748/wjg.v18.i23.3027 PMC3380333

[22] Zeng ZC Tang ZY Fan J Consideration of role of radiotherapy for lymph node metastases in patients with HCC: retrospective analysis for prognostic factors from 125 patients. Int J Radiat Oncol Biol Phys 2005; 63: 1067–76. 15913915 10.1016/j.ijrobp.2005.03.058

[23] Barrowman JA Granger DN. Effects of experimental cirrhosis on splanchnic microvascular fluid and solute exchange in the rat. Gastroenterology 1984; 87: 165–72. 6724260

[24] Thelen A Jonas S Benckert C Tumor-associated lymphangiogenesis correlates with prognosis after resection of human hepatocellular carcinoma. Ann Surg Oncol 2009; 16: 1222–30. 19224279 10.1245/s10434-009-0380-1

[25] Yokomori H Oda M Kaneko F Lymphatic marker podoplanin/D2-40 in human advanced cirrhotic liver—re-evaluations of microlymphatic abnormalities. BMC Gastroenterol 2010; 10: 131. 21059220 10.1186/1471-230X-10-131 PMC2995474

[26] Proulx ST Luciani P Christiansen A Use of a PEG-conjugated bright near-infrared dye for functional imaging of rerouting of tumor lymphatic drainage after sentinel lymph node metastasis. Biomaterials 2013; 34: 5128–37. 23566803 10.1016/j.biomaterials.2013.03.034 PMC3646951

[27] Leijte JA van der Ploeg IM Valdés Olmos RA Visualization of tumor blockage and rerouting of lymphatic drainage in penile cancer patients by use of SPECT/CT. J Nucl Med 2009; 50: 364–7. 19223404 10.2967/jnumed.108.059733

[28] Uenishi T Hirohashi K Shuto T The clinical significance of lymph node metastases in patients undergoing surgery for hepatocellular carcinoma. Surg Today 2000; 30: 892–5. 11059728 10.1007/s005950070040

[29] Bruix J Sherman M. Management of hepatocellular carcinoma: an update. Hepatology 2011; 53: 1020–2. 21374666 10.1002/hep.24199 PMC3084991

[30] Yoon SM Kim JH Choi EK Radioresponse of hepatocellular carcinoma-treatment of lymph node metastasis. Cancer Res Treat 2004; 36: 79–84. 20396570 10.4143/crt.2004.36.1.79 PMC2855105

[31] Park YJ Lim DH Paik SW Radiation therapy for abdominal lymph node metastasis from hepatocellular carcinoma. J Gastroenterol 2006; 41: 1099–106. 17160521 10.1007/s00535-006-1895-x

[32] Wu H Liu S Zheng J Transcatheter arterial chemoembolization (TACE) for lymph node metastases in patients with hepatocellular carcinoma. J Surg Oncol 2015; 112: 372–6. 26368066 10.1002/jso.23994

[33] Utsumi M Matsuda H Sadamori H Resection of metachronous lymph node metastases from hepatocellular carcinoma after hepatectomy: report of four cases. Acta Med Okayama 2012; 66: 177–82. 22525476 10.18926/AMO/48268

[34] Son TQ Hoc TH Long VD Pancreaticoduodenectomy for hepatic portal lymph node metastasis after hepatic resection for hepatocellular carcinoma: a clinical case report. Int J Surg Case Rep 2021; 82: 105921. 33964712 10.1016/j.ijscr.2021.105921 PMC8121700

[35] Yokota Y Tomimaru Y Iwazawa T A resected case of lymph node metastasis at the splenic hilum from lung cancer invading the pancreas and spleen. Jpn J Cancer Chemother 2018; 45: 2208–10. (in Japanese). 30692333

[36] Yilmaz E Celik A. Circumportal pancreas: prevalence, subtypes and vascular variations of 55 patients. Surg Radiol Anat 2018; 40: 407–13. 29380102 10.1007/s00276-018-1975-7

[37] Karasaki H Mizukami Y Ishizaki A Portal annular pancreas, a notable pancreatic malformation: frequency, morphology, and implications for pancreatic surgery. Surgery 2009; 146: 515–8. 19715809 10.1016/j.surg.2009.03.018

[38] Ishigami K Tajima T Nishie A The prevalence of circumportal pancreas as shown by multidetector-row computed tomography. Insights Imaging 2011; 2: 409–14. 22347962 10.1007/s13244-011-0092-5 PMC3259370

[39] Joseph P Raju RS Vyas FL Portal annular pancreas. A rare variant and a new classification. JOP 2010; 11: 453–5. 20818114

[40] Hashimoto Y Ross AS Traverso LW. Circumportal pancreas with retroportal main pancreatic duct. Pancreas 2009; 38: 713–5. 19629006 10.1097/MPA.0b013e3181a910ca

[41] Jang JY Chung YE Kang CM Two cases of portal annular pancreas. Korean J Gastroenterol 2012; 60: 52–5. 22832801 10.4166/kjg.2012.60.1.52

[42] Yamaguchi K Sato N Minagawa N Sarcoidosis in a patient with a circumportal pancreas with a retroportal main pancreatic duct: a case report. Pancreas 2013; 42: 1197–9. 24048464 10.1097/MPA.0b013e31827e2d20

[43] Ohtsuka T Mori Y Ishigami K Clinical significance of circumportal pancreas, a rare congenital anomaly, in pancreatectomy. Am J Surg 2017; 214: 267–72. 27871680 10.1016/j.amjsurg.2016.11.018

[44] Yuan H Wu P Chen J Radical antegrade modular pancreatosplenectomy for adenocarcinomaof the body of the pancreas in a patient with portal annular pancreas, aberrant hepatic artery, and absence of the celiac trunk: a case report. Medicine (Baltimore) 2017; 96: e8738. 29310347 10.1097/MD.0000000000008738 PMC5728748

[45] Kuriyama N Hatanaka T Gyoten K How to divide the pancreatic parenchyma in patients with a portal annular pancreas: laparoscopic spleen-preserving distal pancreatectomy for serous cystic neoplasms. Surg Case Rep 2020; 6: 89. 32358636 10.1186/s40792-020-00852-7 PMC7195503

[46] Abe N Lee SW Shimizu T Surgical management of intraoperatively diagnosed portal annular pancreas: two case reports. Medicine (Baltimore) 2021; 100: e28204. 34918681 10.1097/MD.0000000000028204 PMC8677888

[47] Polyakov AN Mirzaev TS Batalova MV Laparoscopic distal pancreatectomy for portal annular pancreas. Khirurgiia (Mosk) 2023; 6: 108–13. 10.17116/hirurgia2023061108 37313708

[48] Mendoza AS 3rd Han HS Ahn S Predictive factors associated with postoperative pancreatic fistula after laparoscopic distal pancreatectomy: a 10-year single-institution experience. Surg Endosc 2016; 30: 649–56. 26091993 10.1007/s00464-015-4255-1

[49] Pandrowala S Parray A Chaudhari V Portal annular pancreas (PAP): an underestimated devil in pancreatic surgery-systematic review of literature and case report. J Gastrointest Surg 2021; 25: 1332–9. 33555524 10.1007/s11605-021-04927-0 PMC7869770

